# A planetary health–organ system map to integrate climate change and health content into medical curricula

**DOI:** 10.5694/mja2.51737

**Published:** 2022-09-29

**Authors:** Hayden Burch, Laura J Beaton, Grace Simpson, Ben Watson, Janie Maxwell, Kenneth D Winkel

**Affiliations:** ^1^ University of Melbourne Melbourne VIC; ^2^ Centre for Health Policy Melbourne School of Population and Global Health University of Melbourne Melbourne VIC

**Keywords:** Curriculum, Climate change

Health professionals must be prepared to address the health risks and impacts of climate change

Between 2030 and 2050, climate change is expected to cause about 250 000 additional deaths per year.^1^ This does not include deaths from pollution, mental illness, extreme weather events and resultant migration and conflict, all of which carry significant morbidity and mortality risks. The medical profession has a responsibility to prepare practitioners and the health system for the escalating challenges of this health crisis.

Even though planetary health frameworks, consensus statements, and early teaching experience have begun to be reported,^2^, ^3^, ^4^, ^5^, ^6^, ^7^, ^8^, ^9^, ^10^ persistent barriers to the implementation of planetary health concepts, including the health impacts of climate change and the principles of sustainable health care, are evident within medical education.^11^, ^12^ Consequently, the implementation of this content into predominantly biomedically focused curricula is frequently piecemeal, opportunistic, unidirectional and poorly related to clinical practice.^13^, ^14^

Internationally, the emerging curriculum principles to address this challenge align with global health priorities. Specifically, addressing the socio‐economic and environmental determinants of health under the United Nations Sustainable Development Goals framework (https://sdgs.un.org/goals), indigenous eco‐health‐centric leadership, promotion of systems thinking and change, and experiential, practice‐based learning that cultivates interprofessional teamwork, advocacy and leadership development.^3^, ^4^, ^5^, ^6^, ^7^, ^9^, ^10^, ^15^ A 2019 interactive perspective provides a useful open access resource in this direction.^16^ More recently, Emory University students and staff reported their experience in the incorporation of climate content into selected aspects of the pre‐clinical curriculum.^17^

In Australia, Medical Deans of Australia and New Zealand (MDANZ) established a Climate Change and Health Working Group, which has proposed graduate outcome statements and learning objectives that incorporate the environment as a health determinant, health care sustainability, and the health effects of climate change.^18^, ^19^ At the time of publication, the Australian Medical Council is reviewing the accreditation standards of primary medical programs and has released updates to the National Framework for Prevocational Medical Training, strengthening a top‐down national approach to producing climate‐aware and prepared practitioners.

There are many steps between developing new accreditation standards and actual delivery to learners. The three key strategies are: i) integrate climate change as a cross‐cutting core theme, ii) facilitate educators and students sharing knowledge and teaching one another in this emerging field, and iii) relate learning to clinical practice.^11^ An intermediate step is to link existing organ systems focused preclinical studies with the impacts of climate change on the health of individual patients.

## Codevelopment of a planetary health–organ system map

In response to these challenges, students and faculty volunteers, all members of the Doctors for the Environment Australia at the University of Melbourne, collaborated to form the Planetary Health Curriculum Taskforce. This group was driven by student advocacy seeking to address a perceived gap in their curriculum. We contend that such cocreation is essential to accelerate the integration of such urgently needed curriculum content by adopting the highest level of pedagogical methods and to model the intergenerational collaboration required to address this “wicked” challenge.

We sought to achieve three objectives (Box 1) and conducted our work in three phases (Box 2). First, student focus groups from all years of the medical school identified planetary health learning opportunities within the existing curriculum structure.

Box 1 **Methods used to meet project objectives**

**Table:** 

Primary objectives	Methods
Identify current first‐year curriculum alignment with existing Graduate Student Outcomes	Student focus group Review of Graduate Student Outcomes and Intended Learning Outcomes Identification of opportunities for planetary health curriculum enhancement Meeting with the Department of Medical Education regarding opportunities
Exemplify the integration of planetary health as a cross‐cutting theme with particular focus on the mechanistic impacts of climate change on patients and practice; identify and embed shared learning objectives	Meeting with the First Nations health team Meeting with the Australian Medical Students’ Association and the University of Melbourne Medical Students’ Society Consultation with international leaders Ivy Plus Sustainability Consortium (Cornell University) Meeting with Department of Medical Education Literature review via two 5‐hour workshops Expert panel critical review and content validation Synthesis of the meetings, workshops and literature review, expert panel review
Specify opportunities to integrate principles of sustainable health care	Literature review via two 5‐hour workshops Expert panel critical review and content validation Synthesis of the meetings, workshops and literature review, expert panel review

Box 2 **Project phases and outline of creating a planetary health–organ system map**

**Figure:**
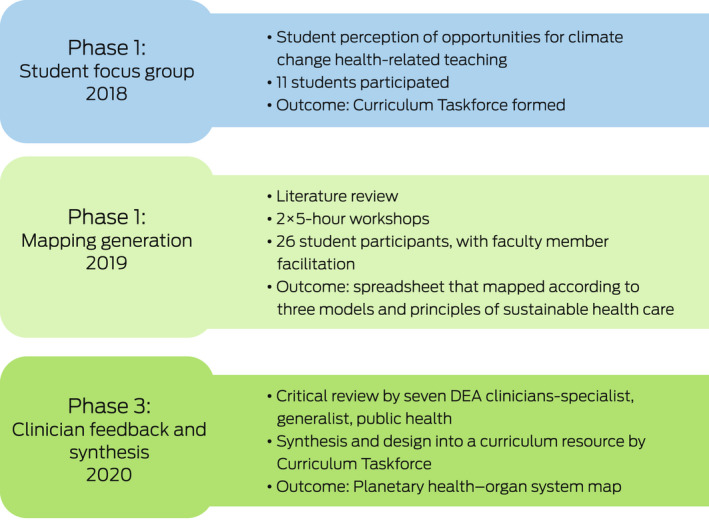
DEA = Doctors for the Environment Australia.

Second, we did a structured literature review, via two student and faculty workshops, to map climate change and health literature content and themes to the classical organ systems framework of first year medicine (Box 3). We incorporated the principles of sustainable health care, such as environmental footprint assessment and valuing the health cobenefits of decarbonisation, as well as the opportunities for medical students to learn applied skills and behaviour. Engagement with the First Nations health team helped us to incorporate Indigenous‐led content, an identified area for further action by Australian and New Zealand public health and medical educators.^12^

Box 3 **The three models used to map the literature on human health and the health of the planet**

**Figure:**
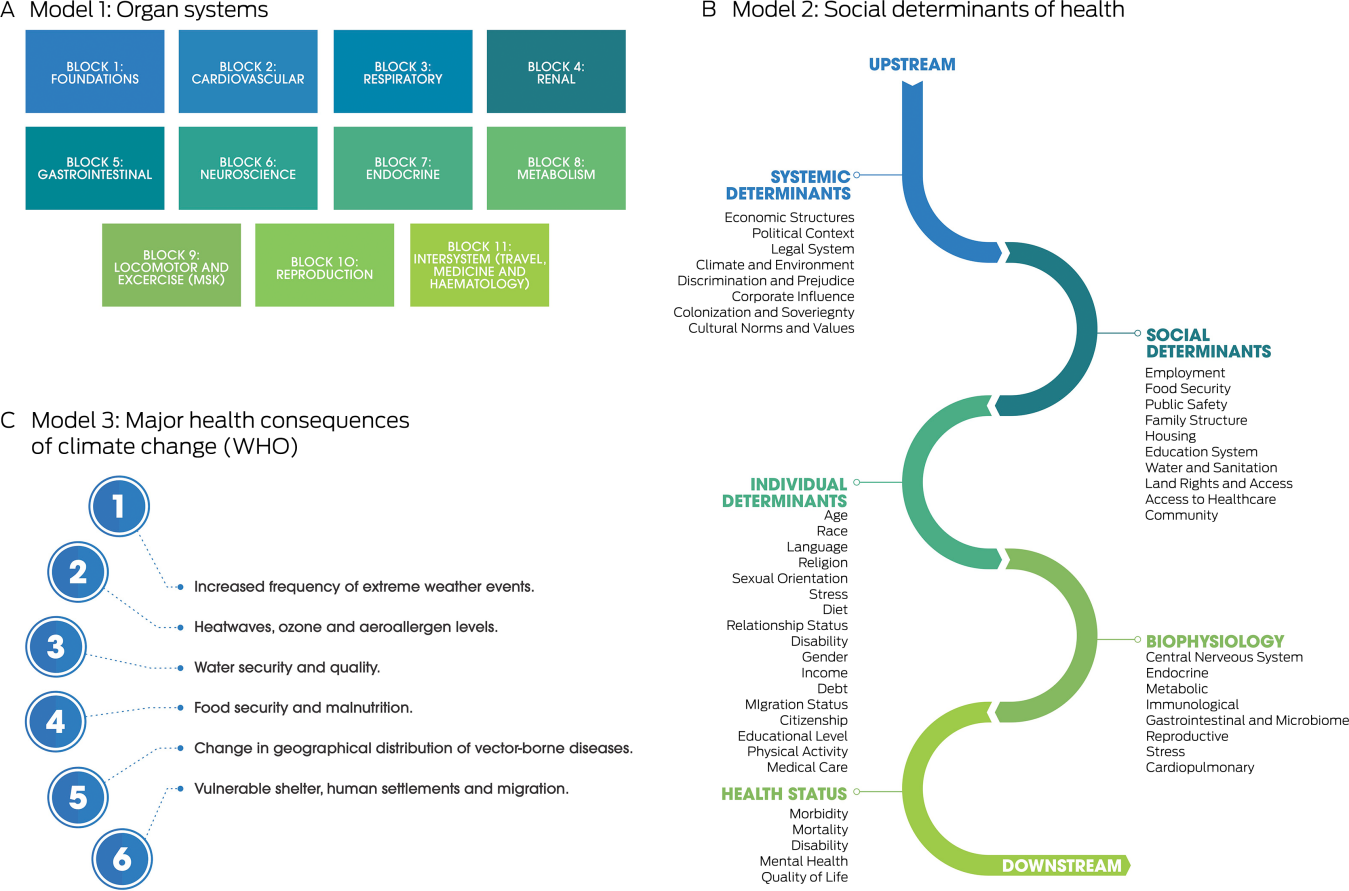
WHO = World Health Organization. Source: Reproduced with permission from Burch et al.^20^

Phase three consisted of expert peer review by seven specialist, generalist and public health clinicians with teaching and research experience in climate change and health. Each clinician was allocated two organ system blocks and instructed to review and edit content for clinical relevance, strength of evidence, and quality of writing.

The final synthesis, inclusion of images, figures and executive summaries were completed by the Curriculum Taskforce student members, with design support from Doctors for the Environment Australia. The outcome was an infographic‐rich curriculum resource, a planetary health–organ system map,^20^ which is being used as a resource in first year medical teaching.

## A guide for climate change integration at all levels of medical education

Our systematic, student and faculty codeveloped process and clinical focus are an example to others seeking successful integration of planetary health into their curricula. Central to our approach was the application of the three selected models (Box 3). This process identified and explored the multidirectional relationships between broad public and planetary health content and person‐centred biophysiological mechanisms. We achieved this by connecting literature content using Model 1 headings (eg, cardiovascular organ system) and Model 2 (relevant social determinants of health) and Model 3 content (relevant major consequences of climate change). As an example, Box 4 shows a mechanism for heatwave‐associated acute myocardial infarction from the planetary health–organ system map.

Box 4 **Example mechanism for heatwave‐associated acute myocardial infarction from the planetary health–organ system map**

**Figure:**
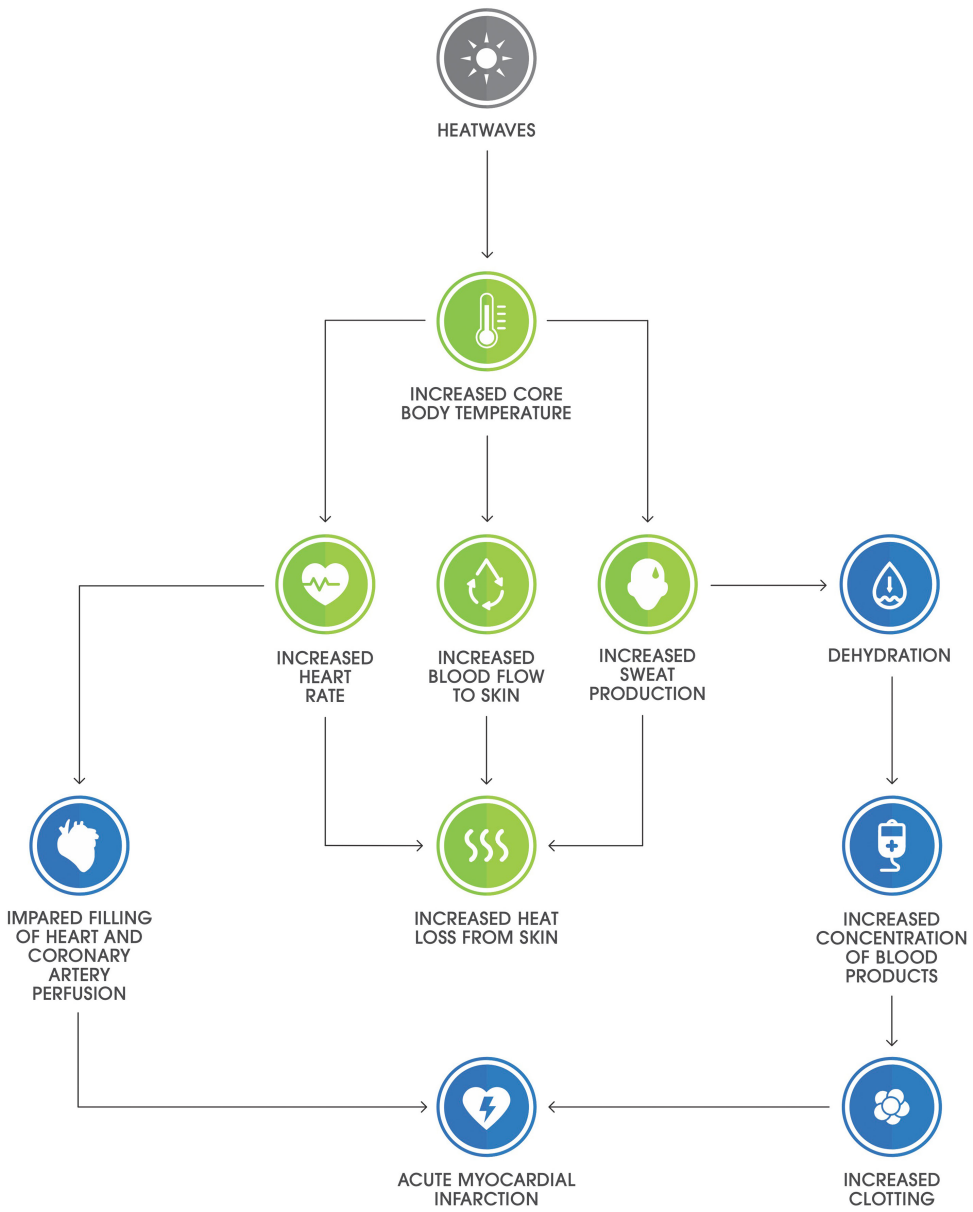
Source: Reproduced with permission from Burch et al.^20^

There is clear recognition by peak Australian medical bodies of the importance of climate change and our professions’ role not just in preparing practitioners, but in advocating for climate‐conscious health care and broader mitigation policies.^21^, ^22^ This has led to an emergent discussion about planetary health at all levels of Australian medical education. Our methodology and output may assist medical educators in addressing the challenges of integrating climate change education into their own teaching.^11^

For example, as this approach enables students to share their knowledge and experiences with teachers outside of formal classroom boundaries, it may support the evident lack of medical educators proficient in planetary health teaching. Empowering students as leaders and partners in learning and developing education material is known to improve their effectiveness as learners and confidence in teaching.^23^ It also explicitly promotes higher order relational and extended abstract reasoning by students (Blooms’ revised taxonomy levels 4–6) — the ultimate task of any curriculum.^24^ Despite not being formally evaluated, we observed that the students involved in this project achieved the Association for Medical Education in Europe (AMEE) planetary health and education for sustainable health care consensus statement aims of “knowledge, skills, values, competence and confidence”.^9^ Our mapping output also addresses the need for learning resources that are centralised, accessible and frequently peer reviewed.

Our focus on clinical relevance helped narrow the scope of literature and developed the curriculum resource towards use in practice. This strategy connected theory with patient care in a way that ensured concepts could be translated into clinical skills. Even though it was designed for one context, the organ systems structure and clinical relevance also allow learning points to be drawn from this resource for prevocational, vocational and continuing professional development teaching.

Our approach also aligns with recent understanding that planetary health is a theme akin to ethics or leadership that should spiral through the core curriculum.^11^ In this context, biomedical planetary health learning primes students for subsequent clinical training.

Our initial successes, including deriving a climate change‐themed tutorial from the map and precipitating a review of planetary health curriculum opportunities within the Doctor of Medicine (MD) program redesign, parallel those of the Emory student–staff collaboration.^17^ The process of expanding the map to cover more of the MD curriculum, regular peer review of emerging evidence and increasing interprofessional collaboration is ongoing and has garnered formal support through seed grant funding.^25^ We encourage the formal recognition of planetary health as core to any medical curriculum, with dedicated resourcing from the top‐down and codesign from the bottom‐up. We recognise and value the concurrent higher level advocacy of the MDANZ Climate Change and Health Working Group in supporting our grassroots project,^18^, ^19^ as well as allyship from our interprofessional and First Nations health colleagues.^25^

A remaining challenge, given the assessment‐driven nature of medicine, is the development of “assessment for learning” inclusive of planetary health. We have not reviewed the assessment blueprint of the University of Melbourne MD program. However, we propose this as a valuable exercise, as assessment often strongly correlates with students’ prioritisation of content.^26^

Ultimately, the role of medical professionals in providing leadership, advocating for sustainable health care and adopting evidence‐based strategies for the management of planetary health‐related risks has been widely acknowledged.^9^, ^21^, ^22^ Our methodology to generate the planetary health–organ system map presents a model for engaging learners through codevelopment of a novel learning resource. Our infographic‐rich map also provides an example of systematic integration with existing curricula and an opportunity for strengthening planetary health leadership in medical education.

## Open access

Open access publishing facilitated by The University of Melbourne, as part of the Wiley ‐ The University of Melbourne agreement via the Council of Australian University Librarians.

## Competing interests

No relevant disclosures.

## Provenance

Not commissioned; externally peer reviewed.
